# Evidence for a Caregiving Instinct: Rapid Differentiation of Infant from Adult Vocalizations Using Magnetoencephalography

**DOI:** 10.1093/cercor/bhv306

**Published:** 2015-12-11

**Authors:** Katherine S. Young, Christine E. Parsons, Else-Marie Jegindoe Elmholdt, Mark W. Woolrich, Tim J. van Hartevelt, Angus B. A. Stevner, Alan Stein, Morten L. Kringelbach

**Affiliations:** 1 Section of Child and Adolescent Psychiatry, Department of Psychiatry; 2 Oxford Centre for Human Brain Activity (OHBA), University of Oxford, Oxford, UK; 3 Center of Functionally Integrative Neuroscience, Department of Clinical Medicine, Aarhus University, Aarhus, Denmark; 4 Department of Psychology; 5 Semel Institute for Neuroscience and Human Behavior, University of California, Los Angeles, CA, USA; 6 Wits/MRC Rural Public Health and Health Transitions Research Unit (Agincourt), School of Public Health, University of Witwatersrand, Johannesburg, South Africa

**Keywords:** caregiving, infant, magnetoencephalography, orbitofrontal cortex, vocalization

## Abstract

Crying is the most salient vocal signal of distress. The cries of a newborn infant alert adult listeners and often elicit caregiving behavior. For the parent, rapid responding to an infant in distress is an adaptive behavior, functioning to ensure offspring survival. The ability to react rapidly requires quick recognition and evaluation of stimuli followed by a co-ordinated motor response. Previous neuroimaging research has demonstrated early specialized activity in response to infant faces. Using magnetoencephalography, we found similarly early (100–200 ms) differences in neural responses to infant and adult cry vocalizations in auditory, emotional, and motor cortical brain regions. We propose that this early differential activity may help to rapidly identify infant cries and engage affective and motor neural circuitry to promote adaptive behavioral responding, before conscious awareness. These differences were observed in adults who were not parents, perhaps indicative of a universal brain-based “caregiving instinct.”

## Introduction

Communication between parents and their offspring has long captured the interest of scientists (Darwin 1872, 1877; Lorenz 1943). From an evolutionary perspective, this early communication is essential for offspring survival by promoting protective and nurturing behaviors in parents. In humans, early parenting behavior is widely acknowledged to have far-reaching consequences for child cognitive and socio-emotional development. Parental responses to infant cries in particular have received much attention as a foundation of attachment relationships (Bowlby 1969; Ainsworth et al. 1978).

Crying, at least in early life, is thought to be largely reflexive, often occurring in response to pain, hunger, or separation from a caregiver (Bell and Ainsworth 1972; Soltis 2004). Much like the solicitation signals of other species (MacLean 1985; Newman 1985), an infant's distress cry ultimately serves to promote proximity between infant and caregiver (Ainsworth 1969; Bowlby 1969; Hunziker and Barr 1986). The sound of a human infant cry is characterized by a high and highly variable pitch, an overall “falling” or “rising–falling” melody, typically with some degree of tremor (or “vibrato”), and often includes abrupt changes in harmonic structure (Kent and Murray 1982; Golub and Corwin 1985). These acoustic features are thought to be largely attributable to infants' short vocal chords and limited muscular control over the vocal tract (Kent and Murray 1982; Ostwald and Murray 1985).

Theoretical models of responsive parental behavior highlight the capacity of vocal and facial cues to capture attention. Lorenz (1943) proposed that the specific configuration of an infant face (“Kindenschema”) acts as an “innate releaser” of caregiving behavior. Building on this theory, Murray (1979) suggested that the acoustic structure of an infant cry acts as a “motivational entity,” rapidly alerting an adult listener, while additional factors contribute to the selection of particular behaviors. Importantly, the motivational entity theory allows for a range of motives for behavior, such as a desire to terminate an aversive stimulus, an empathic response to reduce distress in another, or an evolutionary desire to ensure the wellbeing of offspring (Hoffman 1975; Murray 1979). Observational studies have shown that across cultures, infant crying provokes selective orienting of attention toward the infant and a desire to intervene, typically to provide care (Frodi et al. 1978; Bornstein et al. 2008, 2012). Beyond this initial orienting response, infant-directed behavior can be influenced by a number of other factors including gender, parental experience, specific features of cues, and the broader context of care and culture (Lamb 1977; Wood and Gustafson 2001; Bornstein et al. 2008, 2012; Kruger and Konner 2010).

Adults often report the sound of a crying infant as annoying, distressing, aversive, and likely to promote a desire to perform a caregiving response (e.g., Frodi et al. 1978; Schuetze and Zeskind 2001; Soltis 2004). There is evidence suggesting that hearing infant cries can initiate a broad range of physiological reactions in adult listeners (Frodi et al. 1978; Out et al. 2010). Changes have been demonstrated in heart rate (Frodi and Lamb 1980; Wiesenfeld et al. 1981; Bleichfeld and Moely 1984; Furedy et al. 1989; Del Vecchio et al. 2009), skin conductance (Frodi et al. 1978; Wiesenfeld et al. 1981), blood pressure (Frodi et al. 1978), respiratory sinus arrhythmia (Joosen et al. 2013), hand grip force (Bakermans-Kranenburg et al. 2012), and even skin temperature in breastfeeding mothers (Vuorenkoski et al. 1969). In addition, there is a large body of work showing individual differences in perceptual and physiological reactions to infant cries, varying according to gender, parental status, adult attachment style, and mental health (Furedy et al. 1989; Schuetze and Zeskind 2001; Out et al. 2010; Ablow et al. 2013).

Over the course of the lifespan, audible crying tends to become less frequent with children developing the capacity to inhibit vocalized crying and learning to access care through other, less metabolically costly, means (Bell and Ainsworth 1972; Rebelsky and Black 1972; Gekoski et al. 1983). By adulthood, crying is still a signal of distress, but is less salient to others (Cornelius 1984). Listener's responses to adult cries are largely culturally and contextually determined (e.g., Vingerhoets et al. 2000). Adult crying can alleviate distress by moving the self to action (Tomkins 1962), by establishing physiological homeostasis (Efran and Spangler 1979), or in some cases by motivating others to engage in prosocial behaviors (Hill and Martin 1997; Hendriks et al. 2008). There is little behavioral research directly comparing responses to infant and adult cries; however, we recently demonstrated that adult listeners report a greater motivation to respond to infant compared with adult cries (Parsons, Young, Stein, et al. 2014). At a more implicit level, listening to infant cries can evoke more rapid responding on a motor task, compared with listening to adult cries (Parsons et al. 2012; Young et al. 2015).

The mechanisms by which the brain can process these salient expressions to allow rapid, adaptive behavioral responses are not yet fully understood (Parsons, Stark, et al. 2013). Models of emotional processing describe “dual stream” systems for rapid identification of salient stimuli, followed by slower, detailed appraisal (LeDoux 2000; Adolphs 2002). A number of key regions are recruited in the processing of emotional vocalizations or affective prosody. These include superior temporal sulci/gyri (STS/STG), the orbitofrontal cortex (OFC), the supplementary motor area, middle temporal gyrus, basal ganglia, and amygdala, as confirmed by a recent meta-analysis (Ethofer et al. 2013; Yovel and Belin 2013; Belyk and Brown 2014).

Auditory vocal processing begins with analysis of basic acoustic features in subcortical and primary auditory regions (Belin et al. 2004, 2011; Schirmer and Kotz 2006; Yovel and Belin 2013). Extraction of linguistic, affective, or speaker-related content is then thought to occur within different subregions of the STS/STG (Belin et al. 2004, 2011). The right STS is considered the vocal equivalent of the fusiform face area, a region of the brain that has been shown to be highly selective to the human voice, compared with other environmental sounds (Belin et al. 2000, 2004). This region is particularly sensitive to the affective content of human vocalizations, responding more to emotional, compared with neutral vocalizations (Grandjean et al. 2005). Evidence from EEG studies demonstrates that event-related potentials (ERPs), thought to be generated by activity in the STS/STG (the P200 component), are sensitive to the valence, arousal, and category of emotional vocal stimuli (Paulmann and Kotz 2006, 2008). Supporting this suggestion, fMRI evidence has shown an association between activity in bilateral STS and emotional intensity in vocalizations (Ethofer et al. 2006). Activity in these regions is then thought to project to frontal regions, such as the OFC and IFG for appraisal and higher-order processing [OFC; Schirmer and Kotz 2006; Frühholz and Grandjean 2013; for a review of auditory vocal processing, see Frühholz et al. (2014, 2015)].

The human OFC has been shown to be a nexus for reward-related processing, critically involved in subjective appraisal of stimuli (Kringelbach 2005; Rudebeck and Murray 2014; Berridge and Kringelbach 2015). The temporal unfolding of information processing within this heterogeneous brain region is still under investigation (Kringelbach and Rolls 2004). Studies of individuals with lesions to the OFC have demonstrated impaired recognition of emotional vocal and facial expressions (Hornak et al. 1996, 2003; Grandjean et al. 2008). One such lesion study also demonstrated unaffected sensory processing of emotional vocal stimuli (as shown by the P200 ERP response), suggesting that the OFC may be more involved in evaluative than perceptual processing (Paulmann et al. 2010). In support of this “higher-order” function of the OFC, fMRI studies have demonstrated enhanced activity in this region when task demands require explicit attention for the evaluation of emotional vocalizations (Sander et al. 2005; Quadflieg et al. 2008). There is emerging evidence that the OFC may additionally play a role in the rapid detection of salience. The affective prediction hypothesis suggests that the OFC is involved in early stages of processing, aiding the identification of salient stimuli and facilitating motor reactions (for reviews, see Bar 2003, 2007, 2009). In support, there is mounting evidence that swift propagation of sensory information to the OFC (140 ms) from primary sensory regions may aid preattentive recognition (Bar et al. 2006; Kringelbach et al. 2008; D'Hondt et al. 2013; Parsons, Young, et al. 2013). It has been suggested that feedback projections to sensory areas could then impact further perceptual processing.

The affective prediction hypothesis also suggests that feedforward projections from the OFC to motor areas (e.g., motor cortex and basal ganglia) could prime rapid behavioral responding. One study demonstrated that activity in the OFC and motor cortex was correlated with speed of evaluation of emotional stimuli, supporting this notion (Ethofer et al. 2013). An alternative hypothesis, mirror neuron theory, implicates motor areas more directly in perceptual processing. This theory suggests a neuroanatomical overlap in systems for observing and executing actions (Di Pellegrino et al. 1992; Rizzolatti and Arbib 1998). This early recruitment of the motor system has been strongly emphasized in models of speech perception (Scott and Johnsrude 2003; Watkins et al. 2003; Warren et al. 2006). The role of motor systems in affective vocal processing is less well understood. It has been suggested that the mirror neuron system may support understanding of intention or preparation of imitative or nonimitative motor responses, relevant to the affective content of cues (Leslie et al. 2004; Ferrari et al. 2005; Iacoboni et al. 2005).

Existing studies of neural responses to infant vocalizations point to a comparable network of neural regions to that described above for emotional vocalizations more generally (Lorberbaum et al. 2002; Seifritz et al. 2003; Laurent and Ablow 2012a; Montoya et al. 2012; Riem et al. 2012; De Pisapia et al. 2013; Hipwell et al. 2015). Within this network, enhanced activity in the OFC, as measured with fMRI, has consistently been associated with the processing of multimodal infant cues including vocalizations (e.g., Lorberbaum et al. 2002; Laurent and Ablow 2012a, 2012b) and facial expressions (e.g., Bartels and Zeki 2004; Strathearn et al. 2008; Montoya et al. 2012). Yet, the low temporal resolution and indirect nature of the blood oxygen level-dependent signal in fMRI studies mean that it is very difficult to resolve the fine-grained temporal dynamics of brain activity. A number of recent studies have used magnetoencephalography (MEG) and, in line with the affective prediction hypothesis, there is now evidence that viewing infant faces is associated with greater early activity in the OFC, compared with viewing adult faces (Kringelbach et al. 2008; Parsons, Young, et al. 2013).

Building on these findings of responses to infant faces, we investigated responses to infant vocalizations to assess evidence for early differentiation of infant from adult vocalizations. We used MEG to compare neural activity in response to infant and adult cry vocalizations from a standardized database of emotional vocalizations (the OxVoc database; Parsons, Young, Stein, et al. 2014). We hypothesized that infant and adult cry vocalizations would be associated with differential early activity in auditory, orbitofrontal, and motor cortical regions.

## Materials and Methods

### Participants

MEG was used to examine both the timing and sources of early neural responses to infant and adult cry vocalizations in healthy men and women (*N* = 25, 13 male, 12 female). Participants were aged between 20 and 34 years (M = 23.88, SD = 2.97), and none were parents. They were recruited through email advertisement sent to a pool of participants who had previously participated in MRI research studies at Aarhus University Hospital, Denmark. All participants were right-handed, were not currently taking any medication affecting the brain, and had reported having no hearing problems. Ethical approval for the study was granted by the Ethics Committee of Central Region Denmark.

### Experimental Task

Participants performed a target tone-detection task, within which vocalization stimuli were presented incidentally. The task required participants to make a button press response when they heard a target tone (400 Hz pure tone lasting 500 ms) and not respond when they heard distractor tones (500 Hz pure tone lasting 500 ms; the identity of target and distractor tones was counterbalanced across participants). Fifteen exemplars of each sound type (selected from a larger database, Parsons, Young, Stein, et al. 2014) were presented in each task block. Blocks of each stimulus type were repeated 8 times, resulting in a total of 120 stimulus presentations for each sound category. The order of blocks, and stimuli within blocks, was randomized.

### Stimuli

Sound stimuli consisted of infant and adult cry vocalizations taken from an established stimulus database (Parsons, Young, Stein, et al. 2014). In brief, infant vocalization stimuli were obtained from video recordings of infants interacting with a caregiver in their own homes [see Young et al. (2012)]. During the experimental paradigm, participants also listened to other vocalization stimuli, including neutral and positively valenced infant and adult vocalizations. However, these results are not discussed here. Adult vocalization stimuli were obtained from video diary blogs recorded by females, aged approximately 18–30 years [see Parsons, Young, Stein, et al. (2014) for details]. Permission to use these stimuli for research was obtained directly from parents and individuals involved. Stimuli were presented using Presentation^®^ software (Neurobehavioral Systems, Inc.) using an MEG-compatible in-ear earphone delivery system built in-house.

Stimuli consisted of 1.5 s auditory bursts, matched for root-mean-square intensity and with 150 ms linear rise and fall times applied to each clip. The physical features of infant and adults cry stimuli used are presented in Table 1. Independent *t*-tests demonstrated significant differences in fundamental frequency (*F*_0_) and average burst length, but not in the number of bursts.

**Table 1:** Key physical parameters of vocalization stimuli

Vocalization type	Infant cry		Adult cry		*t*-value	*P*-value	*r*-value
	M, SD	Range	M, SD	Range			
*F*_0_ (Hz)	444.30, 43.16	336.06–527.56	339.84, 64.32	257.81–445.31	5.22	<0.001	0.69
Burst duration (s)	1.06, 0.47	0.45–1.50	0.57, 0.13	0.23–0.75	3.83	0.001	0.58
Number of bursts	1.73, 0.88	1–3	2.13, 0.35	2–3	−1.63	0.12	−0.29

*F*_0_, fundamental frequency.

### Sound Intensity Matching Procedure

Perceived “loudness” of auditory stimuli can impact on the perceived intensity of the emotion within a sound (Murray and Arnott 1993). To match loudness across participants varying in sensitivity to sound intensity, a threshold of hearing test was completed by each participant immediately prior to MEG scanning. An adaptive staircase procedure [comparable to Levitt (1971)] was used to assess individual thresholds of hearing. Briefly, this was a two-alternative forced-choice task in which participants indicated whether they heard sounds of varying intensities. A two-down, one-up design was used, meaning that after a sound was detected, the subsequent sound presentation was decreased by 10 dB, but when a sound was not detected, the subsequent sound presentation was increased by 5 dB. Criterion for absolute threshold of hearing was 2 consecutive “up/down” reversals (the point at which participants report that they could hear a sound, after not hearing the previous sound) at the same value. Stimuli were presented at 70 dB above this level.

### MEG Recordings

MEG recordings were performed using a 306-channel Elekta-Neuromag TRIUX system (Center of Functionally Integrative Neuroscience, Aarhus University Hospital), comprising 102 magnetometers and 204 planar gradiometers at a sampling rate of 1000 Hz. Before recording, a three-dimensional digitizer (Polhemus) was used to record the participant's head shape relative to the position of 5 head position indicator coils fixed to the head. In addition, the positions of 3 fiducial markers (the nasion and the left and right preauricular points) and 20–30 additional scalp “head-shape” points were recorded to aid later coregistration with MRI images. Data were recorded as part of a larger study of auditory processing in back-to-back sessions lasting 15 min each (later concatenated as a single session). A *T*_1_-weighted structural MRI had previously been acquired for each participant as a part of another research study at Aarhus University Hospital (Siemens Trio 3-T scanner, inversion time = 900 ms, time repetition = 1900 ms, time echo = 2.52 ms, flip angle = 9°, slice thickness = 1 mm, field of view = 250 × 250 mm). All MEG data analyses were carried out using an in-house built MATLAB (The MathWorks, Inc.) software with functions from FieldTrip (Oostenveld et al. 2011), SPM8 (Litvak et al. 2011), and FSL (Jenkinson et al. 2012).

#### Data Preprocessing

Channels containing clear artifacts were detected visually and marked as “bad” using downsampled data (sampling rate, 250 Hz) with an in-house built data viewer. External noise was removed using spatiotemporal signal space separation (Taulu and Simola 2006) applied to the raw, non-downsampled data, using only “good” channels (MaxFilter™, Elekta). Cleaned data were then downsampled to 250 Hz and high-pass filtering (0.1 Hz, two-pass Butterworth filter, SPM8) was performed to remove slow drifts in the data. Independent components analysis was used to decompose data into 60 components (data were prewhitened by normalizing to smallest eigenvalues; Hyvarinen and Oja 2000; Vigario et al. 2000). The outputs of this analysis were a number of channels × number of components mixing matrix, 60 temporally independent time courses and their back-projected spatial topographies. Identified components were manually reviewed to remove components containing artifacts (cardiac, ocular, or muscular), based on time courses, frequency spectrum, and spatial topography (Mantini et al. 2011; Muthukumaraswamy 2013). Data were then epoched (250 ms prestimulus to 1750 ms poststimulus) and an in-house automated outlier algorithm was applied, using robust linear regression to detect and remove epochs and channels with outlying minima and standard deviations (epochs with weights <0.40 and channels with weights <0.01 were removed).

#### Sensor-Level (Event-Related Field) Analysis

For event-related field (ERF) analyses, single-trial data were cropped from 200 ms prestimulus to 500 ms poststimulus onset. Data were low-pass filtered at 45 Hz and baseline corrections were performed (using the 100-ms prestimulus period for each trial). Averaged group sensor-level data were used to identify time windows of interest. Sensors exhibiting peak averaged responses during identified time windows in each hemisphere were selected for sensor-level statistical analysis of category-specific effects. Averaged time courses from each individual participant were extracted for each stimulus category and paired *t*-tests (one-tailed) were used to assess differences in ERF amplitude at time windows of interest.

### Source Reconstruction

Preprocessed datasets were concatenated for each participant, taking the first session as a reference for averaging head positions (ensuring a single beamformer solution for each participant; Luckhoo et al. 2014). Anatomical data from *T*_1_-weighted MR scans were segmented and normalized to a template MNI brain. MEG data were coregistered using digitized fiducial markers and refined using additional head-shape points (SPM8).

Source reconstruction was performed using a dual-source adaptation of the Linearly Constrained Minimum Variance (LCMV) beamformer (Van Veen et al. 1997; Woolrich et al. 2011). This method used an overlapping spheres model to estimate dipoles at each location in a 6-mm^3^ mesh representation of the template MNI brain (Huang et al. 1999). Although beamforming has proved powerful at reconstructing source signals in electromagnetic imaging (Hillebrand et al. 2005), it can be limited in the presence of highly correlated source signals, such as early bilateral auditory responses (Van Veen et al. 1997; Sekihara et al. 2002; Herdman et al. 2003; Dalal et al. 2006). To overcome this, a bilateral implementation of the LCMV beamformer was employed, in which the beamformer spatial filtering weights for each dipole were estimated together with the dipole's contralateral counterpart (Brookes et al. 2007). A scalar formulation of the beamformer was used, in which each dipoles' 3 spatial orientations were collapsed to one direction obtained by maximizing the output of the beamformer (Sekihara et al. 2001). This was carried out on both of the bilateral dipoles in the dual-source beamformer. Iterating through all dipole locations in the 6-mm grid yielded a whole-brain estimate of source activity for each trial for each participant in the time window 200 ms prestimulus to 400 ms poststimulus in the frequency range of interest, 1–40 Hz.

### General Linear Model

The source-reconstructed data were epoched into trial-specific time windows of 400 ms (100 ms prestimulus and 300 ms poststimulus), and a separate general linear model was run sequentially at each dipole location for each time point, for each stimulus category (Hunt et al. 2012; see the section “Experimental Procedures” in Supplementary Material). An initial demonstration of beamformer efficacy was carried out using all auditory stimuli from the entire experimental session (*n* = 840), compared against prestimulus baseline (see Supplementary Material). Following resolution of bilateral auditory cortices at 100 ms, differential processing of infant and adult cry vocalizations was assessed. First-level analyses consisted of calculation of “contrast of parameter estimates” (infant cry − adult cry) for each dipole and each time point. Resulting data were converted to absolute values and baseline-corrected using the 100-ms prestimulus period as a baseline. Data were then subject to second-level analyses, using one-sample *t*-tests with variances spatially smoothed with a Gaussian kernel (full-width at half-maximum = 50 mm). Results were computed for a series of *t*-stat maps temporally averaged over 10-ms windows, ranging from 95 to 220 ms poststimulus onset (overlapping by 5 ms). Multiple comparison corrections were conducted using a nonparametric cluster-based permutation test [for details, see Hunt et al. (2012)] with 5000 permutations and a cluster-forming *t*-threshold of 3.2 (equivalent to a corrected *P* < 0.05). Peak coordinates within significant clusters were identified and converted to *z* statistics.

## Results

### Behavioral Data

Performance during the tone-detection task was at ceiling level with no significant differences in reaction times to the tones during the blocks of presentation of infant and adult cries (*t*_(24)_ = 0.05, *P* = 0.86; mean RT = 691 ms).

### Summary of Results of MEG Data Analysis

We used standard Matlab tools to analyze the MEG data of all 25 participants both in sensor space and at the source level. Sensor-level analyses demonstrated significant differences in ERF time courses at both 100 and 200 ms to infant cries compared with adult cries in the right hemisphere. Using source-level analysis, we found early (at 95–135 ms) significant differences in activity between infant and adult cry vocalizations in temporal (STG and temporal pole) and frontal regions (OFC and anterior cingulate cortex [ACC]), and later differences (175–205 ms) in visual and motor regions.

### Sensor-Level Analysis

**Figure 1.**
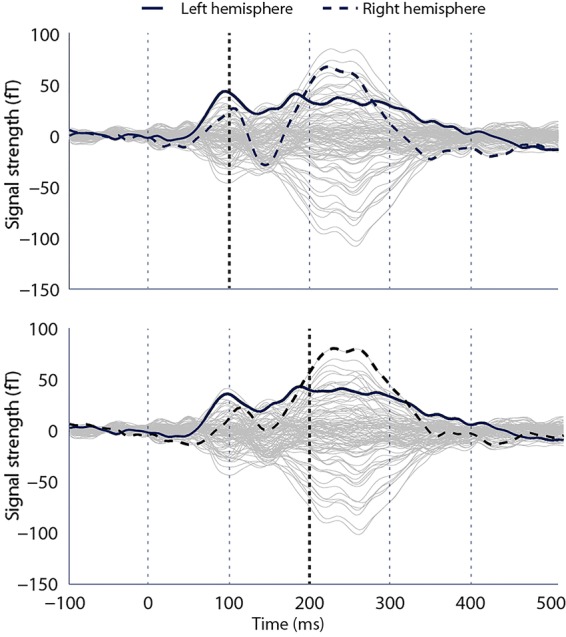
Individual sensor time courses (averaged across conditions) presenting maximally responding channels at 100ms (upper) and 200ms (lower). Vertical dashed lines indicate timing intervals, and lines in “bold” demonstrate time point analyzed.

**Figure 2.**
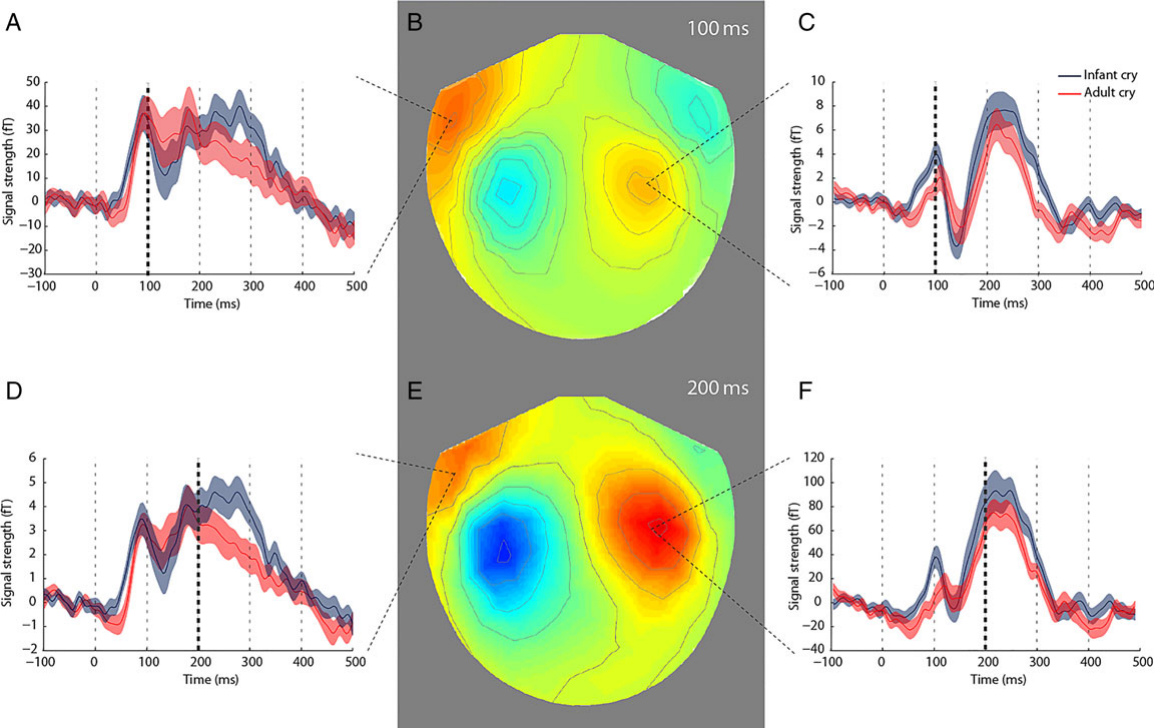
Sensor-level data showing averaged ERFs at 100 ms (*B*) and 200 ms (*E*) poststimulus onset. (*A*, *C*, *D*, and *F*) Category-specific time courses from peak sensors in each hemisphere from −100 ms prestimulus to 500 ms poststimulus. In the right hemisphere only, the amplitude of ERFs from peak sensors were greater in response to infant cries (blue) than to adult cries (red) at 100 ms (*P* < 0.05) and 200 ms (*P* = 0.06). Error bars represent mean ± standard error. Vertical dashed lines indicate timing intervals, and lines in “bold” demonstrate time point analyzed.

Figure 1 shows the averaged ERF time courses in response to all auditory stimuli (combined across conditions and participants) for each MEG channel. Based on these ERFs, time windows of interest were identified as the classical early auditory ERF components (the N100 and P200). Maximally responsive channels at each of these time points in each hemisphere (4 channels in total) were selected for further analysis. Time courses from each of these sensors are presented in Figure 2, along with scalp topographies. Paired-sample *t*-tests demonstrated greater ERF amplitude in response to infant cries, compared with adult cries that was significant at 100 ms [*t*_(24)_ = 1.97, *P* = 0.03, *r* = 0.37] and approached significance at 200 ms [*t*_(24)_ = 1.64, *P* = 0.06, *r* = 0.32] in maximally responsive channels in the right hemisphere. There were no significant differences in ERF amplitude in maximally responsive channels in the left hemisphere at 100 ms [*t*_(24)_ = 0.22, *P* = 0.41, *r* = 0.04] or 200 ms [*t*_(24)_ = −0.63, *P* = 0.27, *r* = 0.13]. Right hemisphere channels showing significant differences were fronto-temporally located (Fig. 2).

### Source-Level Analysis

Source reconstruction demonstrated different spatial distributions of neural activity in response to infant and adult vocalizations from 90 to 200 ms poststimulus (significant at cluster level *P* < 0.05, corrected for multiple comparisons using nonparametric cluster-based permutation tests, see the Methods section for details). Table 2 presents peak coordinates within significant clusters demonstrating differential early activity to infant and adult cries.

**Table 2:** Neural activity across poststimulus time windows differentiating infant and adult cry vocalizations

Time window (ms)	Cortical region	*t*-stat	MNI coordinates			L/R
			*X*	*Y*	*Z*	
90–100	Primary auditory cortex (STG)	4.38	42	−30	12	R
95–105	Auditory cortex	4.14	42	−30	18	R
100–110	Primary auditory cortex (STG)	4.54	54	−30	18	R
105–115	Primary auditory cortex (STG)	5.59	54	−30	18	R
110–120	Primary auditory cortex (STG)	6.54	60	−24	12	R
115–125	Primary auditory cortex (STG)	7.60	60	−24	12	R
120–130	Primary auditory cortex (STG)	8.34	60	−24	12	R
	Primary auditory cortex (STG)	4.07	−60	−24	6	L
125–135	Primary auditory cortex (STG)	8.15	60	−24	12	R
	Primary auditory cortex (STG)	4.24	−42	−24	24	L
	Orbitofrontal cortex	4.47	−34	20	−16	L
	Temporal pole		−46	18	−22	L
	Anterior cingulate cortex		−6	36	18	L
130–140	Primary auditory cortex (STG)	4.93	−54	−30	12	L
	Primary auditory cortex (STG)	7.4	54	−24	12	R
135–145	Primary auditory cortex (STG)	6.62	54	−24	12	R
	Primary auditory cortex (STG)	4.96	−54	−30	12	L
140–150	Auditory cortices	5.74	60	−24	18	R
	Primary auditory cortex (STG)	4.59	−54	−30	12	L
145–155	Auditory cortices	4.42	60	−24	18	R
	Auditory cortices	3.97	−60	−18	18	L
150–160	Auditory cortices	4.64	−60	−18	18	L
155–165	Auditory cortices	4.64	−66	−18	18	L
	Primary auditory cortex (STG)	4.09	68	−18	0	R
160–170	Primary auditory cortex (STG)	4.32	68	−18	0	R
165–175	Primary auditory cortex (STG)	4.41	68	−18	6	R
170–180	Primary auditory cortex (STG)	4.37	−60	−24	12	L
175–185	Primary auditory cortex (STG)	4.35	−60	−24	12	L
	Motor cortex		−46	−8	36	L
180–190	Auditory cortices	4.20	−36	−18	36	L
185–195	Auditory cortices	4.13	−30	−24	18	L
	Visual cortex	4.08	12	−84	−6	R
190–200	Auditory cortices	3.89	−60	−18	18	L
	Orbitofrontal cortex	3.83	−34	20	−16	L
	Temporal pole		−36	24	−24	L
	Anterior cingulate cortex	3.87	18	18	30	R

Note: Peak voxels within clusters of significant activity are reported, with thresholding at *P* < **0**.05 (corrected for multiple comparisons), listed by time point and cortical region. There were no significant differences observed from 195–205, 200–210, 205–215, or 210–220 ms.

**Figure 3.**
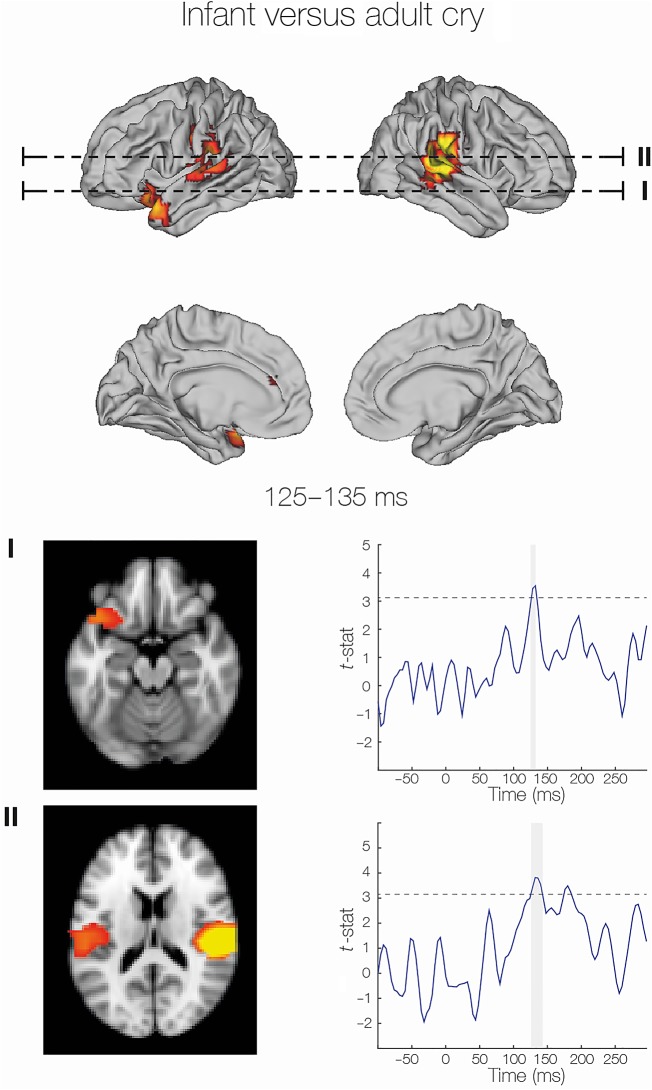
Listening to infant compared with adult cry vocalizations was associated with differential early transient activity in the OFC peaking around 130ms after stimulus onset. During this time, differential auditory cortex activity is also at a peak. Source reconstruction of the infant versus adult cry contrast is demonstrated (upper) and axial slices at the levels shown are demonstrated (lower). Statistical differences over time (*t*-stat time courses derived from the general linear model) are also presented to demonstrate the transient nature of these effects. Dotted lines indicate a *t*-threshold of 3.2 (equivalent to *P* < 0.05), and vertical gray bars indicate the time that differential activity is above this threshold.

**Figure 4.**
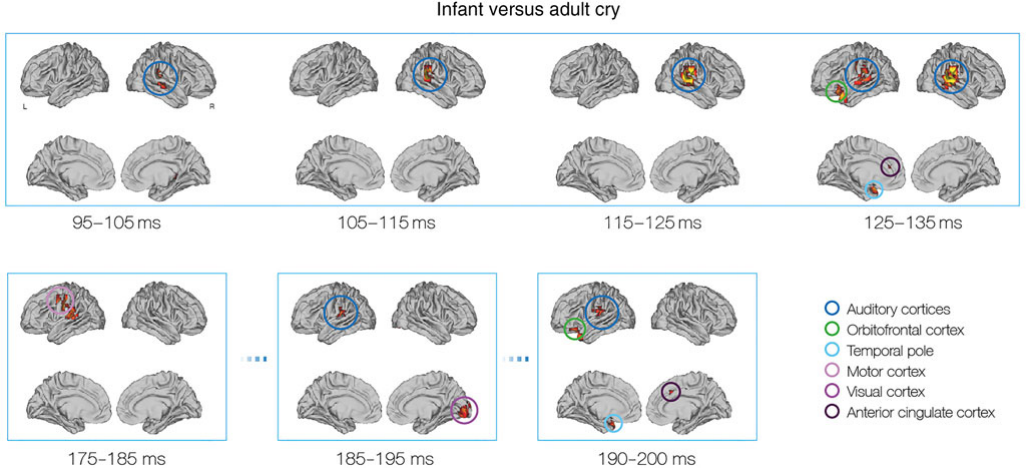
The evolution of differential neural activity in response to infant and adult cry vocalizations from 95 to 200ms. Time windows demonstrating changes of interest are presented (statistical details using overlapping 10ms windows are presented in Table 1). Early differences were observed in right-lateralized auditory regions. At 125–135 ms, there were differences in the OFC and anterior cingulate cortex (ACC). Later differences were demonstrated in the motor and visual cortices, with repeat recruitment of OFC and ACC at 195–205ms. Data were rendered onto inflated brain templates using the Connectome Workbench v1.0 tool (Marcus et al. 2011).

To summarize, the earliest differences in neural activity in response to infant and adult cry vocalizations were observed at around 100 ms. These differences were apparent in auditory regions, and were particularly strong in the right STG (see Fig. 3 for spatial distribution and Table 2 for statistics and coordinates of significant clusters). Differential processing in auditory regions continued throughout the time window tested (95–200 ms). From 125 to 135 ms, a transient difference in frontal activity was observed in the left OFC and anterior cingulate cortex (Fig. 4). Following this, differential processing moved to more posterior locations in the brain, including the postcentral gyrus (145–165 ms), the motor cortex (175–185 ms), and visual cortex (185–195 ms). Finally, from 190 to 200 ms, a second transient burst in frontal activity was observed, also localized to the OFC and anterior cingulate cortex.

## Discussion

We demonstrated rapid differentiation of infant and adult vocal distress in auditory, emotional, and motor brain regions. Differential activity, at a speed faster than conscious processing (Sergent et al. 2005), was found at the source level in emotional regions (OFC and ACC, 125–135 and 190–200 ms) and motor regions (175–185 ms). We also found sustained neural activity differentiating these stimuli in auditory regions (90–200 ms). This was corroborated by sensor-level findings of enhanced ERF activity for infant compared with adult cry vocalizations at 100 ms poststimulus in right-lateralized MEG sensors.

An infant's cry carries particular salience for adult listeners, signaling need for care and promoting the initiation of caregiving behavior (Lorenz 1943; Murray 1979). Previous work has demonstrated that adults have specific behavioral and psychophysiological responses to these sounds (e.g., Parsons et al. 2012; Joosen et al. 2013). Here, we show evidence for how this might be supported by early differential neural activity. The timing of this activity, in the range of 100–300 ms, is considered “preconscious” in studies that demonstrate a typical delay of around 500 ms between the onset of neural activity and the emergence of mental awareness [most notably described and examined by Libet (2002, 2006)].

We observed sustained differential activity in “voice-selective” STS/STG, thought to be sensitive to intensity, type, and salience of vocal emotion (STS/STG; Belin et al. 2004, 2011; Ethofer et al. 2013). In our data, early differences (90–120 ms) were observed in the right hemisphere only. This lateralization has previously been conceptualized as important in assessing the spectral content of auditory stimuli [for review, see Schirmer and Kotz (2006)]. Beyond 120 ms, we showed differential activity in both left and right auditory cortices, in line with recent work demonstrating bilateral processing of affective prosody (Frühholz and Grandjean 2013; Frühholz et al. 2015). The current study was not designed to investigate the functional roles of different components of activity. However, we would hypothesize that, in line with a large body of previous work, early differential activity in STS/STG is likely related to perceptual, rather than evaluative, processes. As infant and adult cry stimuli differ in pitch, it is plausible that early auditory cortex activity reflects processing of this acoustic feature. Future work could aim to investigate this by using stimuli that systematically vary in acoustic properties.

The OFC is a key region involved in the appraisal of emotional expressions across modalities (Adolphs 2002; Hornak et al. 2003). Theories of auditory emotional processing suggest involvement of the OFC at around 300 ms poststimulus, after perceptual processing has occurred in auditory regions (Schirmer and Kotz 2006). Our data partially support this, demonstrating earlier STG than OFC activity for differentiating vocal emotions. However, we found evidence of 2 transient bursts of activity in the OFC, occurring earlier than 300 ms (at 125–135 and 190–200 ms). We suggest that this is more consistent with the affective prediction hypothesis which proposes a dynamic interplay between OFC and sensory regions that may enable preattentive recognition of salient stimuli and prime behavioral responses (Bar 2007; Barrett and Bar 2009).

The neurobiology of parent–infant interactions is thought to heavily recruit networks involved in reward processing and social cognition, of which the OFC is thought to play a central role (Parsons et al. 2010; Swain et al. 2011; Parsons, Stark, et al. 2013; Feldman 2015). We have previously demonstrated early, differential OFC activity in response to infant faces compared with adult faces (Kringelbach et al. 2008; Parsons, Young, et al. 2013). Findings presented here show a similar effect in the auditory domain. Taken together, we speculate that this reflects a neural representation of a “caregiving instinct”, a preconscious response that may allow rapid detection and evaluation of infant-specific cues. Critically, these effects were observed in adults who were not parents, suggesting a potentially universal, modality-independent sensitivity to infant cues in the OFC. Replication and more detailed investigation of this effect for infant vocalizations would be important for exploring this hypothesis further. In the visual domain, we previously demonstrated diminished OFC responses to infant faces when there was a structural abnormality (cleft lip), suggesting that this early response relies on particular configurations of stimulus features (Parsons, Young, et al. 2013). Similar work characterizing changes in OFC responses relating to varying stimulus properties, task demands, and behavioral performance would be important for investigating the extent of this effect in the auditory domain. Given the wealth of extant research demonstrating specific behavioral and physiological responses to infant cries, future work combining multiple levels of analysis (e.g., neural, physiological, and behavioral) in the same individuals would also be an important future avenue for research into human caregiving behavior.

We also observed activity in the ACC from 190 to 200 ms, similar to previous fMRI studies that have demonstrated ACC activity in response to infant cry vocalizations, compared with artificial control stimuli (e.g., Lorberbaum et al. 2002; Sander et al. 2007; De Pisapia et al. 2013). Here, we show that this region is also sensitive to the difference between infant and adult cry vocalizations. The role of the ACC in caregiving behavior has previously been highlighted in animal studies, showing that ACC lesions can disrupt rodent maternal responses to pup vocalizations (e.g., Murphy et al. 1981). More broadly, the ACC in humans has been conceptualized as an “alarm system,” mediating signals communicating both physical and social pain (Eisenberger and Lieberman 2004). Within this context, emotional vocalizations from conspecifics could constitute “social pain” stimuli, communicating distress in others and perhaps aiding the orienting of attention.

Differences in neural activity were also found in the motor cortex. Previous studies using fMRI to investigate neural responses to infant vocalizations have not robustly demonstrated a role for motor cortical regions, although one study demonstrated motor cortex activity in response to infant faces (Caria et al. 2012). Findings presented here show that differential motor cortex activity was transient in nature and therefore unlikely to be detected with the slower temporal resolution of fMRI. Both the affective prediction hypothesis and mirror neuron theory suggest early involvement of motor regions in the processing of affective vocalizations (as highlighted in motor theories of speech perception; Rizzolatti and Arbib 1998; Scott and Johnsrude 2003). Further work investigating the functional role of this activity would be of much interest.

### Potential Limitations

MEG provides greater sensitivity to cortical than subcortical sources of neural activity (Hillebrand and Barnes 2002). As such, there is limited power to detect differential activity in subcortical regions, such as the amygdala and basal ganglia, regions thought to support rapid processing and evaluation of emotional stimuli (e.g., LeDoux 2000; Dolan 2002; Paulmann et al. 2008). Previous work has also demonstrated differential acoustic processing in the auditory brainstem (Kraus and Nicol 2005; Nan et al. 2015) and differential sensitivity to infant cry vocalizations in the periaqueductal gray of the midbrain (using intracranial recordings; Parsons, Young, Joensson, et al. 2014). Evidence from techniques more sensitive to subcortical regions, such as fMRI, is important to consider in neural models of affective auditory processing. This is of particular importance in the context of a proposed “caregiving instinct” as these processes are likely to have been evolutionarily conserved and as such may recruit phylogenetically older (e.g., subcortical) brain structures. In addition, the current paradigm involved incidental listening, so we are unable to directly specify what impact this differential processing may have on behavior.

### Future Directions

Investigating other types of motivationally salient auditory cues would further assess the extent to which early OFC functioning is comparable across modalities. Similarly, inclusion of relevant behavioral measures would inform theories of the function of this rapid differential activity. Previous work investigating individual differences in behavioral and physiological responses to infant cry vocalizations have demonstrated a number of effects including differences related to participant sex, parental status, depressive symptoms, and attachment style (Furedy et al. 1989; Schuetze and Zeskind 2001; Out et al. 2010; Ablow et al. 2013). The current study was not sufficiently powered to look at these differences, but future work should investigate the neural correlates of these previously demonstrated effects. Specific to the further investigation of human parenting behavior, assessment of these effects in parents of young infants would allow investigation of the impact of parental experience on these processes. In addition, identification of neural activity specific to the recognition of one's own infant would further inform our understanding of the neural underpinnings of the parent–infant relationship. Linking neural activity to caregiving behavior over time would eventually provide a better mechanistic understanding of the brain changes associated with the transition to parenthood.

## Conclusions

We demonstrate evidence for rapid differentiation of infant and adult distress vocalizations in auditory, emotional (OFC), and motor brain regions. We propose that hearing an infant cry initiates dynamic activity among these brain regions that may aid quick detection and evaluation of these salient cues and prime adaptive responding. Taken together with previous findings demonstrating similarly early OFC activity in response to infant faces, we suggest that these responses may form part of the neural basis of a so-called “caregiving instinct”. Future work investigating how this neural signature may change as a result of parental experience, and in relation to caregiving behavior, would be of particular interest.

## Supplementary Material

Supplementary material can be found at: http://www.cercor.oxfordjournals.org/.

## Funding

The research was supported by an MRC Studentship awarded to K.S.Y., an ERC Consolidator Grant: CAREGIVING (no. 615539), and a TrygFonden Charitable Foundation grant to M.L.K, a Wellcome Trust Grant (no 090139) to A.S. as well as the Center for Music in the Brain at Aarhus University. Funding to pay the Open Access publication charges for this article was provided by the Wellcome Trust.

## Supplementary Material

Supplementary Data
