# Supplementary material for: Evidence for a Caregiving Instinct: Rapid Differentiation of Infant from Adult Vocalizations Using Magnetoencephalography
Source: Cereb Cortex. 2015 Dec 11;26(3):1309–21. doi: 10.1093/cercor/bhv306 (PMC4737615; doi:10.1093/cercor/bhv306)
Supplement: Supplementary Data [file supp_26_3_1309__index.html]

Evidence for a Caregiving Instinct: Rapid Differentiation of Infant from Adult Vocalizations Using Magnetoencephalography — Supplementary Data 

# Evidence for a Caregiving Instinct: Rapid Differentiation of Infant from Adult Vocalizations Using Magnetoencephalography

## Supplementary Data

Supplementary Data

- Supplementary Data - Docx file
