# Supplementary material for: Evidence for a Caregiving Instinct: Rapid Differentiation of Infant from Adult Vocalizations Using Magnetoencephalography
Source: Cereb Cortex. 2015 Dec 11;26(3):1309–21. doi: 10.1093/cercor/bhv306 (PMC4737615; doi:10.1093/cercor/bhv306)
Supplement: Supplementary Data [file supp_bhv306_bhv306supp.docx]

**Supplemental Data**

*Reliability of the bilateral LCMV beamformer*

Figure S1 shows the basic contrast of all auditory stimuli at 100ms compared to a pre-stimulus silent baseline period (-100ms to 0ms). The bilateral LCMV beamformer demonstrated reliable reconstruction of bilateral auditory cortices in response to auditory stimuli.


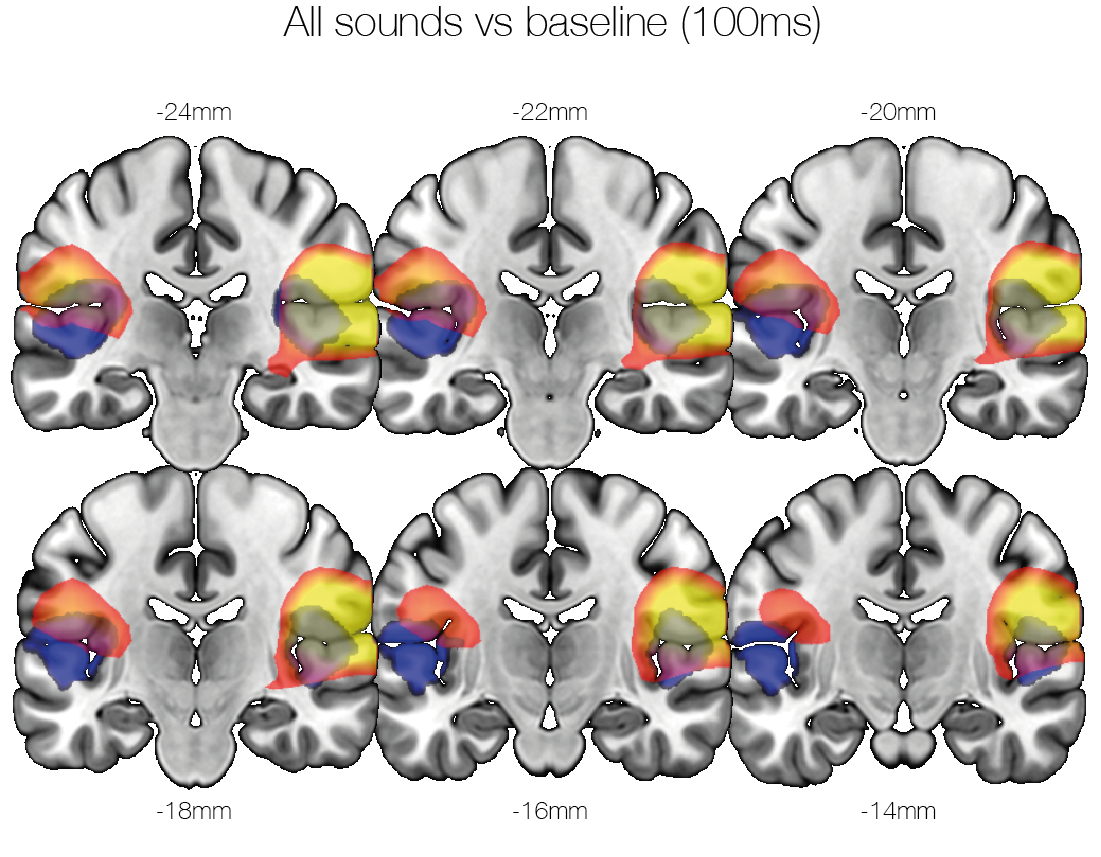


Figure S1. Activity across all sounds vs baseline at 100ms (red-yellow) and conjunction of bilateral TE1.0, TE1.1 and TE1.2 maps from the Juelich atlas (in blue), superimposed on the 0.5mm^3^ MNI atlas. Auditory activity thresholded at *t* > 5.

**Supplemental Experimental Procedures – GLM implementation**

A General Linear Model (GLM) ([Hunt et al., 2012](#_ENREF_1)) was used to resolve evoked responses in source-reconstructed data. The source-reconstructed data, d, was epoched (100 ms pre-stimulus to 300 ms post-stimulus), and a separate GLM was run sequentially at each dipole location, r_i_, for each time-point, t, within trials, tr, over which the experimental stimuli varied:

(1)

where X denotes the design matrix linking each trial to the stimulus category; *β* contains the parameter estimates (PEs) for each category for each time point; and *ε* is the error term of the model.

Two contrasts were performed at the first-level: C_main_ summing all PEs (i.e. the main effect of listening to sounds), and C_diff_ subtracting PEs of *infant cry* and *adult cry* (i.e. the difference between listening to infant cries and adult cries). This produced two contrast parameter estimates (COPEs; CPE­­_mains­_ *_­_*and CPE_diff_) for each dipole location and each time-point for each participant. Absolute values of CPE_mains_ and CPE_diff_ were baseline corrected (using a -100 ms to 0 ms pre-stimulus window) and submitted to a group-level analysis. The use of absolute values accounts for the sign ambiguity of source dipoles across participants at the first level. This ambiguity occurs as the dipole orientation maximizing the beamformer output (as described under **Source-reconstruction**) is unlikely to be the same across participants, and the sign of the COPE at a given dipole location and time point in one participant may be reversed in another.

A one-sample *t*-test was used to evaluate the group effect for each dipole location, r_i_, and time point, t. This analysis yielded a group-level version of CPE_mains_ and CPE_diff_, g-CPE_mains_ and g-CPE_diff_ respectively, along with their between-participant variances, var_mains_ and var_diff_ estimated using ordinary least-squares regression. These variances were subsequently smoothed spatially with a Gaussian kernel (FWHM = 50 mm) and *t*-stats were calculated as:

 (2)

 (3)

The *t*-stat map of g-CPE_mains_ was generated to test the reliability of resolving bilateral responses in the auditory cortices (see Supplemental Data). To investigate differential responses to infant and adult cry vocalisations, temporally averaged *t*-stat maps were computed for 10 ms windows within the time-range of interest (95-220ms post-stimulus, 5 ms overlap, 25 images in total). Nonparametric cluster based permutation testing was performed on the whole brain g-CPE_diff_ *t*-stat map ([as introduced in Hunt et al., 2012](#_ENREF_1)).

For each time window 5000 permutations of the averaged *t*-stat data were run, using a cluster-forming *t*-threshold of 3.2 (FSL **randomise** function). The size of the largest cluster produced by each permutation was used to build a null-distribution of cluster sizes. To get *p*-values corrected for multiple comparisons over space, the same threshold (3.2) was used on original time-averaged *t*­-stat maps and resulting cluster sizes were compared with those of the null-distribution. Significant clusters, surviving a corrected *p*-value of 0.05 were identified (FSL **cluster** function) and used within each time window as a mask for the original averaged *t*-stat maps (FSL **fslmaths** function).
